# Genomic insights into two new subspecies of Herbaspirillum huttiense strains isolated from diseased foliage in Florida

**DOI:** 10.1099/ijsem.0.006597

**Published:** 2024-12-13

**Authors:** Mousami Poudel, Anuj Sharma, Gerald V. Minsavage, Kiersten Fullem, Jose Huguet-Tapia, David J. Norman, Erica M. Goss, Carrie L. Harmon, Jeffrey B. Jones

**Affiliations:** 1 Plant Pathology Department, University of Florida, Gainesville, FL, USA; 2 Mid-Florida Research and Education Center, University of Florida, Apopka, FL, USA; 3 Emerging Pathogens Institute, University of Florida, Gainesville, FL, USA; 4 Plant Diagnostic Clinic, University of Florida, Gainesville, FL, USA

**Keywords:** ANI, bacterial taxonomy, *Herbaspirillum*, T3SS

## Abstract

The genus *Herbaspirillum* comprises 13 species, the majority of which are plant colonizers. However, some species are occasionally isolated from environmental sources, including water and polluted soil, while others are opportunistic human pathogens. Four novel bacterial strains were isolated from diseased foliage of tomato and Boston fern in Florida, USA. Phylogenetic analysis based on the 16S rRNA gene sequence placed all strains into the genus *Herbaspirillum*. The Gram-negative strains produced opaque, creamy white, mucoid colonies, which is typical of the genus *Herbaspirillum*. Biolog biochemical profiling also identified those strains as members of *Herbaspirillum*. The strains were subjected to whole-genome sequencing, and their genomes were compared with those of reference strains of *Herbaspirillum* spp. using average nucleotide identity (ANI). The two strains isolated from Boston fern shared 99% pairwise ANI, as did the two strains isolated from tomato. Among all reference genomes tested, the novel strains shared the highest ANI to *Herbaspirillum huttiense* subsp. *huttiense* (G21-1742 and NC 40101, 96.76%; SE1, 97.23%; F1, 97.16%) and to *H. huttiense* subsp. *putei*. These values are above the established 95% threshold for species delineation based on ANI. As the ANI between members of the two currently described subspecies of *H. huttiense*, i.e. *huttiense* and *putei*, is also ~97%, it can be inferred that the two groups of novel strains described in this study should be considered as candidates for classification as two new subspecies of *H. huttiense*, given that the current *H. huttiense* subspecies also have ~97% with the fern and tomato strains. *In silico* DNA–DNA hybridization results were consistent with those of ANI; comparison of G21-1742 and NC 40101 with *H. huttiense* subsp. *putei* IAM 15032^T^and *H. huttiense* subsp. *huttiense* LMG 2199^T^ produced DNA–DNA hybridization (DDH) values of 66.1 and 73.6 %, respectively. Similarly, SE1 and F1 had DDH values of 68.9 and 68.8% with *H. huttiense* subsp. *putei* IAM 15032^T^ and 77.1 and 76.7% with *H. huttiense* subsp. *huttiense* LMG 2199^T^, respectively. The genomes of all novel isolates carry genes involved in plant pathogenesis, including those of the type III secretion system, which are not present in other *H. huttiense* strains. Based on genomic and phenotypic data, we conclude that these strains represent the first phytopathogenic subspecies within *H. huttiense* and the names proposed are *H. huttiense* subsp. *nephrolepidis* for the two strains isolated from *Nephrolepis exaltata* (designated strain, G21−1742=LMG 33362=NCPPB 4765) and *H. huttiense* subsp. *lycopersici* (designated strain, SE1=LMG 3361=NCPPB 4764) for the two strains isolated from *Solanum lycopersicum*.

## Data Availability

The whole-genome sequences of the strains G21-1742, NC 40101, SE1 and F1 have been deposited in GenBank under accession numbers JAVLSM000000000, JBCGUI000000000, JAVLSJ000000000 and JAVRAB000000000, respectively. The nucleotide sequences obtained in this study were deposited into the GenBank database under the following accession numbers for 16S rRNA (G21-1742, OR004801.1; NC 40101, PP848216; SE1, EF216332.1; F1, EF216331).

## Introduction

The genus *Herbaspirillum* was first proposed in 1986 [1] and currently includes 13 species. Most *Herbaspirillum* species are plant colonizers, while others have been reported from polluted soil, wells and groundwater, as well as acting as opportunistic human pathogens [2,3]. *Herbaspirillum* species are often documented as epiphytes, endophytes or plant pathogens in members of the *Poaceae* family [3]. The species *Herbaspirillum seropedicae* has been isolated from rice (*Oryza sativa*), maize (*Zea mays*), sorghum (*Sorghum bicolor*) and *Sachharum* hybrids and has been found to provide beneficial effects to its host plants, acting as plant growth-promoting rhizobacteria through various mechanisms including N_2_ fixation [1]. *Herbaspirillum rubrisubalbicans* causes stripe disease of sorghum (*S. bicolor*) as well as mottled stripe disease in some susceptible varieties of sugarcane (*Sachharum oficinarum*) [4,5]. Red stripe disease of sorghum is characterized by red streaks alongside the secondary leaf veins, particularly close to the site of inoculation. Although adversely impacting the lifespan of infected leaves [4,6], the disease does not have a significant effect on crop productivity [7]. The type III secretion system (T3SS) in *H. rubrisubalbicans* is essential for the development of the mottled stripe disease in sugarcane and for endophytic colonization of rice [5].

*Herbaspirillum huttiense* is a species that has been documented to inhabit environmental niches. It was initially described in 2004 when it was isolated from well water [8]. The species currently contains two recognized subspecies, *H. huttiense* subsp. *huttiense* and *H. huttiense* subsp. *putei* [9]. In the early 2000s, the strains of non-fluorescent non-pigmented bacteria were isolated from 3- to 4-week-old tomato transplant seedlings in a commercial greenhouse in Florida and were identified as members of the *Herbaspirillum* genus based on 16S rRNA analysis [10]. In October of 2021, additional bacterial strains, most closely related to *H. huttiense*, were isolated from the pinnules of Boston fern (*Nephrolepis exaltata*) from a nursery operation in Florida [11]. In both cases, disease symptoms were mild and consisted of tan leaf spots and minor blighting. In this study, we sought to identify these novel strains using phenotypic, chemotaxonomic and comparative genomic analyses.

## Isolation and growth of bacteria

The two strains, G21-1742 and NC 40101, were isolated from diseased Boston fern leaf samples at the Plant Diagnostic Center, University of Florida (Gainesville, FL) and Plant and Pest Diagnostic Lab, North Carolina State University (Raleigh, NC), respectively [11]. The strains SE1 and F1 were isolated from symptomatic tomato foliage from greenhouse seedling production in Florida [10]. The source of bacterial strains was stored at −80 °C in 30% glycerol amended with 0.8% nutrient broth solution. The strains were streaked on nutrient agar (NA) (Difco™, Becton Dickinson and Co, MD) and incubated at 28 °C prior for 24 h in all experiments. Bacterial colonies of all four strains appeared opaque, creamy white, mucoid and round with smooth margins. The isolates were Gram-negative, oxidase-positive, aerobic, non-pectolytic and non-fluorescent on King’s Medium B. Pathogenicity tests of all four strains were confirmed in their respective hosts of origin in previous studies [10,11]. The strains were deposited in international bacterial culture collections with the accession numbers G21-1742 (LMG 33362=NCPPB 4765) and SE1 (LMG 3361=NCPPB 4764). Initial identification of the strains was done using 16S rRNA phylogenetic analysis (Fig. S1, available in the online Supplementary Material) as described by [11] and also Type (strain) Genome Server (TYGS) [12,13] 16S rRNA gene analysis (Fig. S2), which grouped the isolates closest with *H. huttiense* and two other *Herbaspirillum* spp.

## Phenotypic characterization

Phenotypic characterization was carried out with the Biolog GENIII Microplate (Biolog, Inc., Hayward, CA). Bacterial cultures were grown overnight on Biolog Universal Growth agar (Biolog, Inc., Hayward, CA). A small amount of bacterial cells was then suspended in Biolog inoculation fluid at 95% turbidity (optical density = 0.022), and 100 µl of the suspension was added to each well of the GENIII Microplate and incubated at 28 °C for 48 h according to the manufacturer’s protocol. The results were recorded manually. The comparison of the resulting profiles was done with the Biolog database, which identified all four strains as *H. huttiense*. The Biolog results of the type strain *H. huttiense* subsp. *huttiense* LMG 2199^T^ were compared with those of the proposed new subspecies and are disclosed in Table S1.

## Matrix-Assisted Laser Desorption/Ionization Time-of-Flight Mass Spectrometry (MALDI-TOF MS)

Pure cultures of the bacterial strains (one from each group: G21-1742 and SE1) were grown overnight on NA plates, which were then sent to Charles River Laboratories (Newark, DE, USA) for analysis using MALDI-TOF MS. Resulting spectra were compared to the spectra of known species contained within Bruker Biotyper (v. 11758) and Charles River MALDI-TOF MS libraries (v. 23.01) for identification. The identification results from the MALDI-TOF MS indicated that the top species match for representative strains of both fern (G21-1742) and tomato (SE1) is *H. huttiense* with scores of 2.110 and 2.130, respectively. The range of score values for probable species identification is 1.75–3.0.

## Transmission electron microscopy

**Fig. 1.**
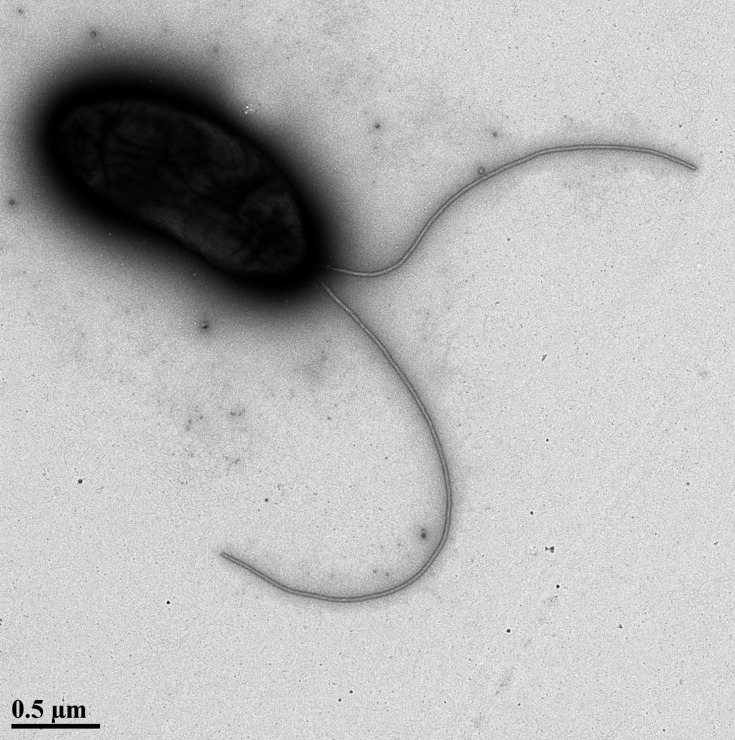
Transmission electron microscopy of strain G21-1742 shows a slightly curved rod with two polar flagella.

Transmission electron microscopy (TEM) was performed at the Interdisciplinary Center for Biotechnology Research, University of Florida (UF ICBR), using a Tecnai G2 Spirit TWIN 120 kV Transmission Electron Microscope. Through this analysis, strain G21-1742 was revealed to possess a slightly curved rod-shaped cell morphology and lophotrichous flagellar arrangement (Fig. 1).

## Genomic characterization

Wizard Genomic DNA Purification Kit (Promega, Madison, WI) was used for genomic DNA extraction for the four novel *Herbaspirillum* strains. DNA was sent to the SeqCenter (Pittsburgh, PA, USA) for whole-genome sequencing using the Illumina NextSeq 2000 platform. The resulting sequences were assessed for quality using FASTQC software (v. 0.11.7). Genome data processing and analyses were carried out using the HiPerGator3.0 supercomputer at the University of Florida. Reference strains utilized for computational analyses were obtained from the publicly available databases of the National Center for Biotechnology Information. The assembly of draft genomes for all strains was accomplished using the procedure outlined by Timilsina *et al*. [14]. Sequence contigs were *de novo* assembled using the assembly program SPAdes V.3.10.1 [15]. CheckM (v. 1.1.2) was employed to assess the quality of draft genome assemblies utilizing the *Herbaspirillum* genus-level taxonomic marker set [16]. Assembled sequences and selected *Herbaspirillum* reference genomes were functionally annotated using Prokka (v. 1.14.6) with default parameters for bacteria [17]. Genomes of strains G21-1742 and NC 40101 were ~6.42 and 6.44 Mbp, respectively, and comprised 65 and 59 contigs. Similarly, genomes of strains SE1 and F1 were ~6 and 5.88 Mbp, respectively, and comprised 45 and 35 contigs. High-quality draft genomes of strains G21-1742, NC 40101, SE1 and F1 were deposited in GenBank under accession numbers JAVLSM000000000, JBCGUI000000000, JAVLSJ000000000 and JAVRAB000000000 (Table 1).

**Table 1.** Genome statistics of the strains sequenced in this study

Bacterial species	Strain	Source of isolation	GenBank account no.	No. of contigs	Size (bp)	G+C content (%)	*N*_50_ (bp)
*H. huttiense* subsp. *nephrolepidis*	G21-1742	*N. exaltata*	JAVLSM000000000	65	6 425 485	61.88	336 392
*H. huttiense* subsp. *nephrolepidis*	NC 40101	*N. exaltata*	JAVRAA000000000	59	6 445 839	61.85	323 225
*H. huttiense* subsp. *lycopersici*	SE1	*S. lycopersicum*	JAVLSJ000000000	45	6 007 189	62	382 859
*H. huttiense* subsp. *lycopersici*	F1	*S. lycopersicum*	JAVRAB000000000	35	5 882 573	62	452 892
*H. huttiense* subsp. *huttiense*	LMG 2199^T^	Distilled water	JBDHMT000000000	41	5 445 188	62.83	257 163

The type strain LMG 2199T was sequenced in this study.

**Fig. 2.**
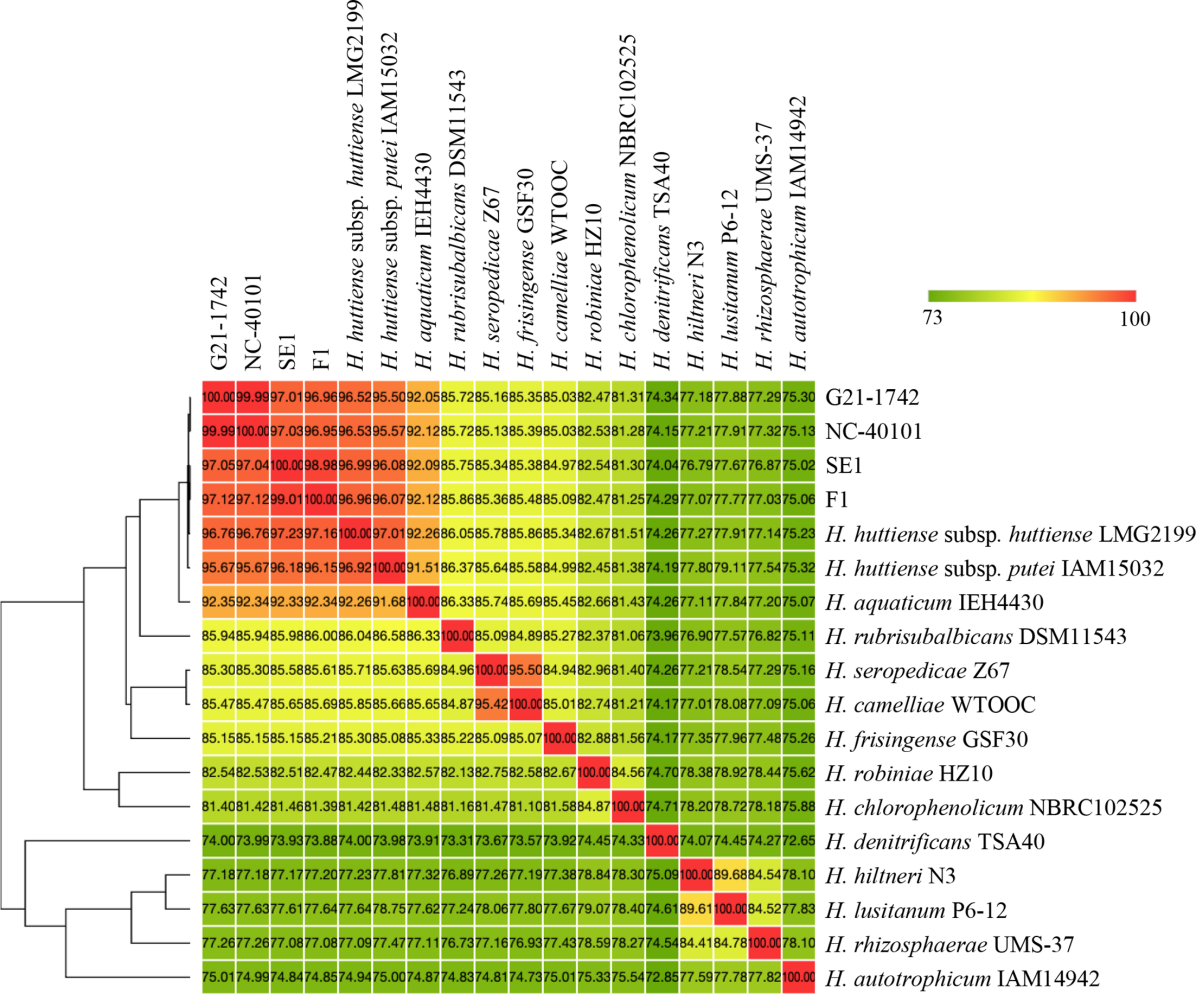
ANI values between the whole-genome sequences of the four novel strains (G21-1742, NC 40101, SE1 and F1) and representative species of *Herbaspirillum*.

Average nucleotide identity based on blast (ANIb) was calculated using Pyani (v.0.2.10) [18,19]. Average nucleotide identity (ANI) comparisons between various *Herbaspirillum* strains used in this study (Table 1) showed near-identical ANI values of 99% between fern strains (G21-1742 and NC 40101) and between tomato strains (SE1 and F1), signifying a very high level of genomic similarity (Fig. 2). The ANI values between strains G21-1742, NC 40101, SE1 and F1 when compared to *H. huttiense* type strain LMG 2199^T^ were ~97% which is above the commonly accepted threshold (typically 95% or higher) for defining strains as belonging to the same species [19]. Additionally, ANI values between fern and tomato strains were found to be ~97% (Fig. 2). As the ANI values between members of the two currently described subspecies of *H. huttiense,* i.e. *huttiense* and *putei*, is also ~97%, it can be inferred that the two groups of novel strains described in this study should be considered as candidates for classification as two new subspecies of *H. huttiense*. This is supported also by their isolation from distinct hosts. *In silico* DNA–DNA hybridization (*is*DDH) values were calculated using formula 2 of Genome-to-Genome Distance Calculator v3.0 [12], and the *is*DDH results were consistent with those of ANIb (Table 2). The comparison of G21-1742 and NC 40101 with *H. huttiense* subsp. *putei* IAM 15032^T^and *H. huttiense* subsp. *huttiense* LMG 2199^T^ had produced DNA–DNA hybridization (DDH) values of 66.1 and 73.6%, respectively. Similarly, SE1 and F1 showed DDH values of 68.9 and 68.8% with *H. huttiense* subsp. *putei* IAM 15032^T^ and 77.1 and 76.7% with *H. huttiense* subsp. *huttiense* LMG 2199^T^, respectively. Our findings suggest that these strains represent the first phytopathogenic subspecies within *H. huttiense*. For the subspecies isolated from Boston fern (*N. exaltata*, strains G21-1742 and NC 40101), we propose the name *H. huttiense* subsp. *nephrolepidis*. For the subspecies isolated from tomato (*Solanum lycopersicum*, strains SE1 and F1), we propose the name *H. huttiense* subsp. *lycopersici*.

**Table 2.** *is*DDH values (in %) between the strains and type strains of *H. huttiense* subsp. *putei* and *H. huttiense* subsp. *huttiense* using TYGS comparison server using the second formula

Strains		1	2	3	4	5	6
G21-1742	1	100	100	76.6	76.3	66.1	73.6
NC 40101	2	100	100	76.6	76.3	66.1	73.6
SE1	3	76.6	76.6	100	92.4	68.9	77.1
F1	4	76.3	76.3	92.4	100	68.8	76.7
*H. huttiense* subsp. *putei* IAM 15032^T^	5	70.12	66.1	68.9	76	100	74.3
*H. huttiense* subsp. *huttiense* LMG 2199^T^	6	73.6	73.6	77.1	76.7	74.3	100

**Fig. 3.**
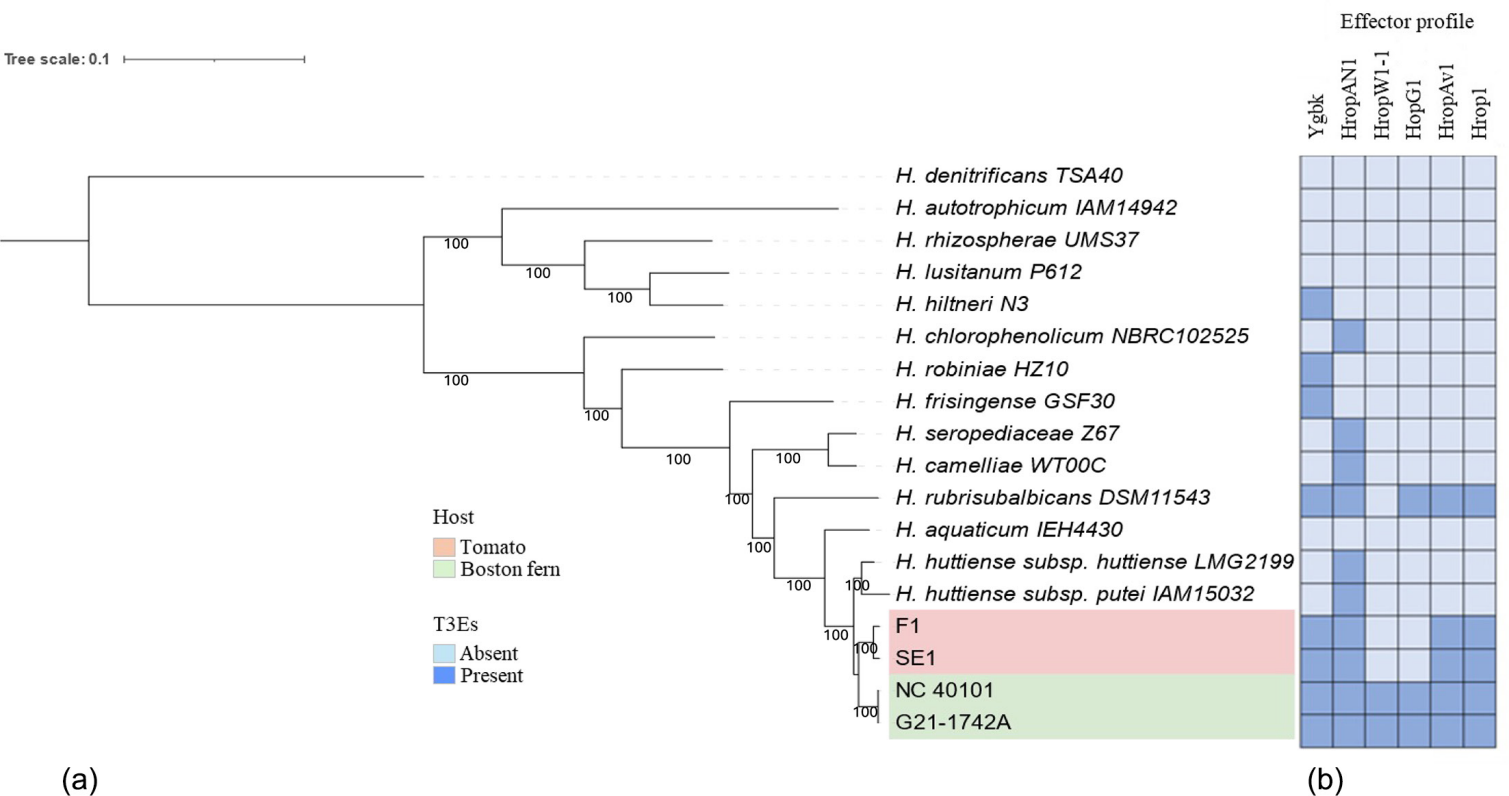
(a) Maximum likelihood phylogenetic tree with 100 bootstrap values of *Herbaspirillum* spp. based on 1666 core genes of tomato (red), fern (green) and 13 type strains of different *Herbaspirillum* spp. (black).(**b**) T3Es with the presence/absence variation were determined based on a threshold of >60 % amino acid identity covering >30 % of query length.

For core genome phylogeny, the gene content of the strains used in this study was compared using Roary (v.3.12.0) [20]. Core genes were defined as genes present in 100% of the strains. Maximum likelihood phylogenetic analysis was conducted using RAxML (v 8.2.10) with the GTR GAMMA I substitution model [21], and the resulting phylogenetic trees were visualized using Interactive Tree Of Life (iTOL) (v.5.6.3)[22]. A total of 1666 core genes were identified using Roary from the four strains sequenced in this study along with the annotated genomes of the representative type strains of 13 *Herbaspirillum* spp. The core genome phylogenetic analysis (Fig. 3a) placed tomato (SE1, F1) and fern (G21-1742, NC 40101) strains into two distinct clades which together were sister to a clade containing the previously defined subspecies of *H. huttiense* (*H. huttiense* subsp. *huttiense* strain LMG 2199^T^ and *H. huttiense* subsp. *putei* strain IAM 15032^T^). Furthermore, the genomes of novel isolates were submitted to the TYGS) or additional validation of phylogenetic clusters. TYGS generates a whole genome-based phylogenetic tree by comparing the submitted genomes with the top ten closely related type strains from its database, establishing species clusters [12,13]. In the TYGS computed whole-genome phylogeny, the fern and tomato strains formed sister clades and branched closest with *H. huttiense* (Fig. S3). These results provide further support for the classification of tomato and fern strains as separate subspecies within the *H. huttiense* complex.

## Comparative analyses of T3SS genes and effectors and nitrogen fixation genes

**Fig. 4.**
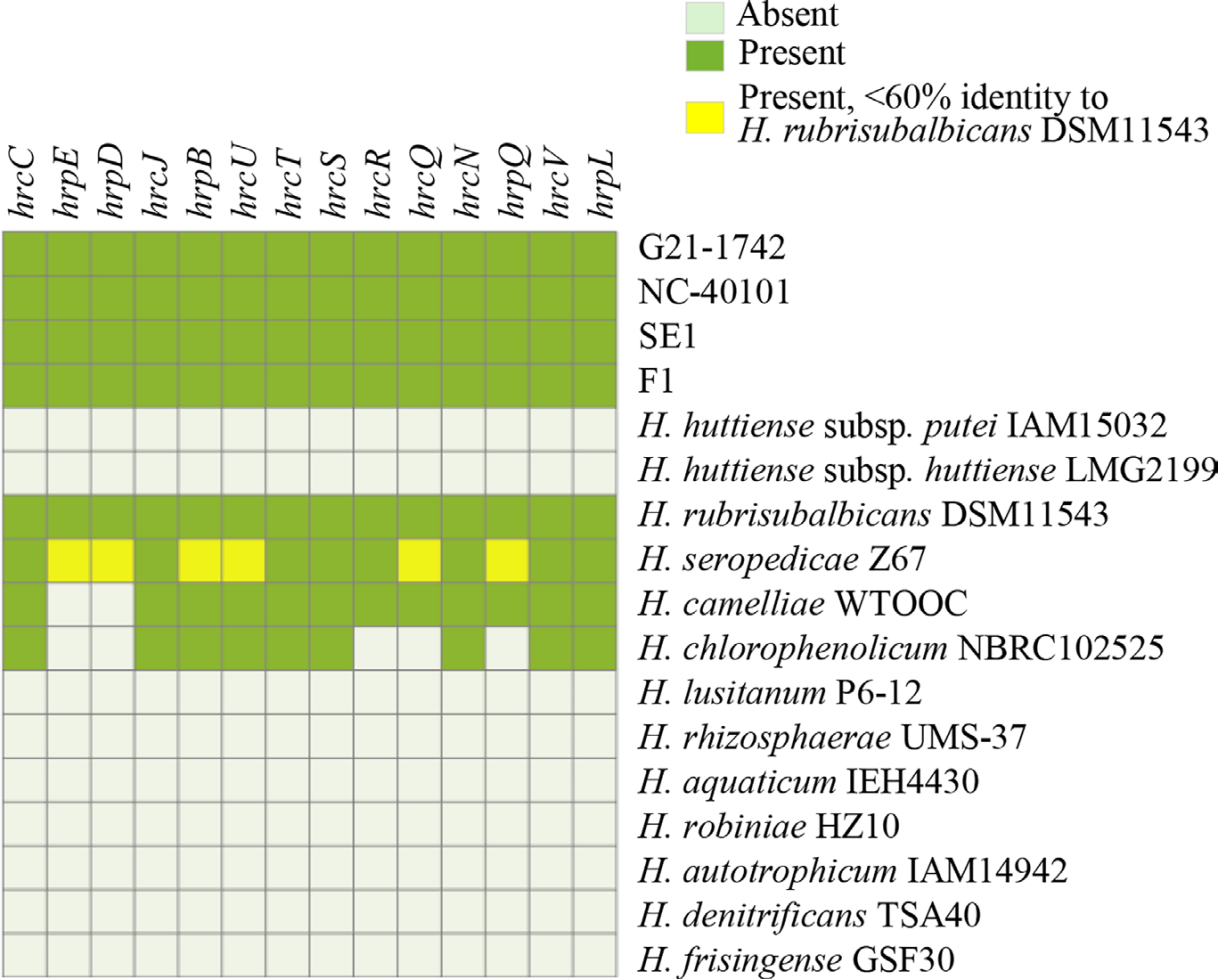
Comparison of T3SS genes in the strains sequenced in this study and type strains of the genus *Herbaspirillum*. Effectors with presence/absence variation were determined based on a threshold of >60 % amino acid identity covering >50 % of query length.

**Fig. 5.**
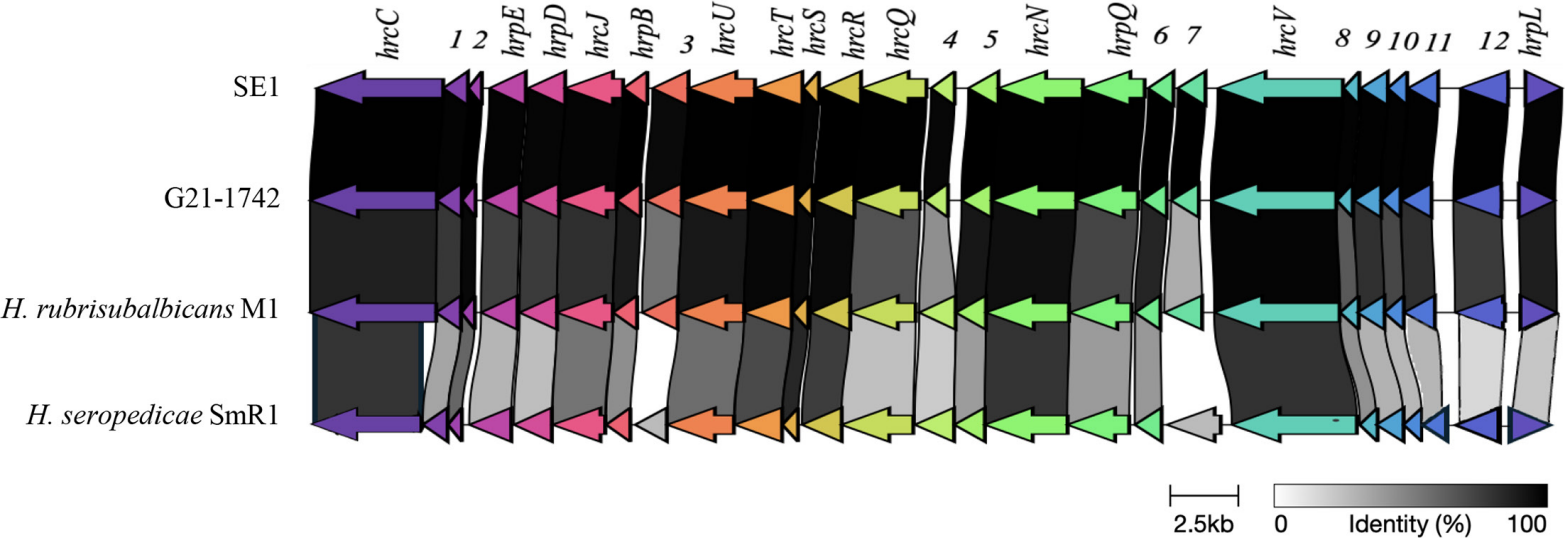
Genetic organization of T3SS of the fern strain (G21-1742), tomato strain (SE1)*, H. rubrisubalbicans* strain M1 and *H. seropedicae* strain SmR1. Homologous genes are shown in the same colours.

Previously characterized T3SS and type III effector (T3E) genes of *H. rubrisubalbicans* strain M1 were used as query sequences in tBLASTn analysis to identify T3SS and T3E genes or homologs in the novel strains [5]. Only hits that had ≥60% identity and ≥30% query coverage were considered to indicate the presence of an effector. To identify and visualize T3SS homologous gene clusters among the genomes, Comparative Gene Cluster Analysis Toolbox (CAGECAT), a clinker software, was used [23]. The fern (G21-1742 and NC 40101) and tomato (SE1 and F1) strains contained all 14 of the T3SS genes present in *H. rubrisubalbicans* strain M1 (Fig. 4) as well as 12 additional gene sequences encoding hypothetical proteins, all distributed within the 21 kb region containing T3SS genes. Comparison of the DNA sequences of the *hrp/hrc* cluster of the tomato and fern strains with *H. rubrisubalbicans* strain M1 showed that the genes are under synteny (Fig. 5). However, the *hrp/hrc* cluster of fern and tomato strains had lower percentage identity with *H. seropedicae* strain SmR1, which suggests that the fern and tomato strains might have acquired their *hrp/hrc* genes from * H. rubrisubalbicans* strain M1 (Table S2).

The fern and tomato strains sequenced in this study exhibited similar T3E profiles to the plant pathogenic *H. rubrisubalbicans* strain M1 (Fig. 3b). Notably, the protein HropAN1 (3-oxo-tetronate kinase) showed complete identity between tomato and fern strains and shared 96.73% identity with 100% coverage with the T3E protein HopAN1 from plant pathogenic bacterium *Burkholderia fungorum*. It has been reported that HopAN1 is present in both plant pathogenic strains such as *Pseudomonas syringae* pv. *syringae* strain B728a and the non-T3SS encoding symbionts *Methylobacterium mesophilicum* strain SR1 and *Erwinia billingiae* strain Eb661 [24]. Similar is the case with HropAN1 in the genus *Herbaspirillum* as it is present in both pathogenic *H. rubrisubalbicans* and symbiotic diazotroph *H. seropedicae* and *H. huttiense* subsp. *huttiense*. This effector gene’s presence in a diverse range of both T3SS and non-T3SS symbiotic bacteria suggests a functional role beyond type III secretion, extending to non-pathogenic bacteria.

Similarly, HropAV1 from fern and tomato strains had 73% identity and 100% coverage with the HropAV1 effector from *H. rubrisubalbicans* strain M1 and showed homology (52.66% identity and 95% query coverage) with a T3E protein from plant pathogenic bacteria *Ralstonia solanacearum*, known to promote growth in eggplant [25]. HropW1-1, unique to fern strains, shared homology (52.53% identity and 81% query coverage) with a *P. syringae* effector involved in disrupting the actin cytoskeleton to enhance virulence in *Arabidopsis* [26]. Fern strains also harboured HopG1, which shared homology with the XopAG effector in *Xanthomonas citri* pv. *citri*, known to induce cell death in grapefruit leaves [27].

Hrop1 proteins encoded in fern and tomato strains demonstrated high similarity with *H. rubrisubalbicans* strain M1 (79.18% identity with 99% coverage). It has been reported that Hrop1 is homologous to a T3E protein from *R. solanacearum* strain MolK2 [5]. However, the presence of the T3E protein YgbK was observed in tomato and fern strains, along with other species like *H. rubrisubalbicans*, *Herbaspirillum robinae* and *Herbaspirillum frisingense*, sharing the highest similarity with *H. rubrisubalbicans* (91% identity and 96% query coverage).

**Fig. 6.**
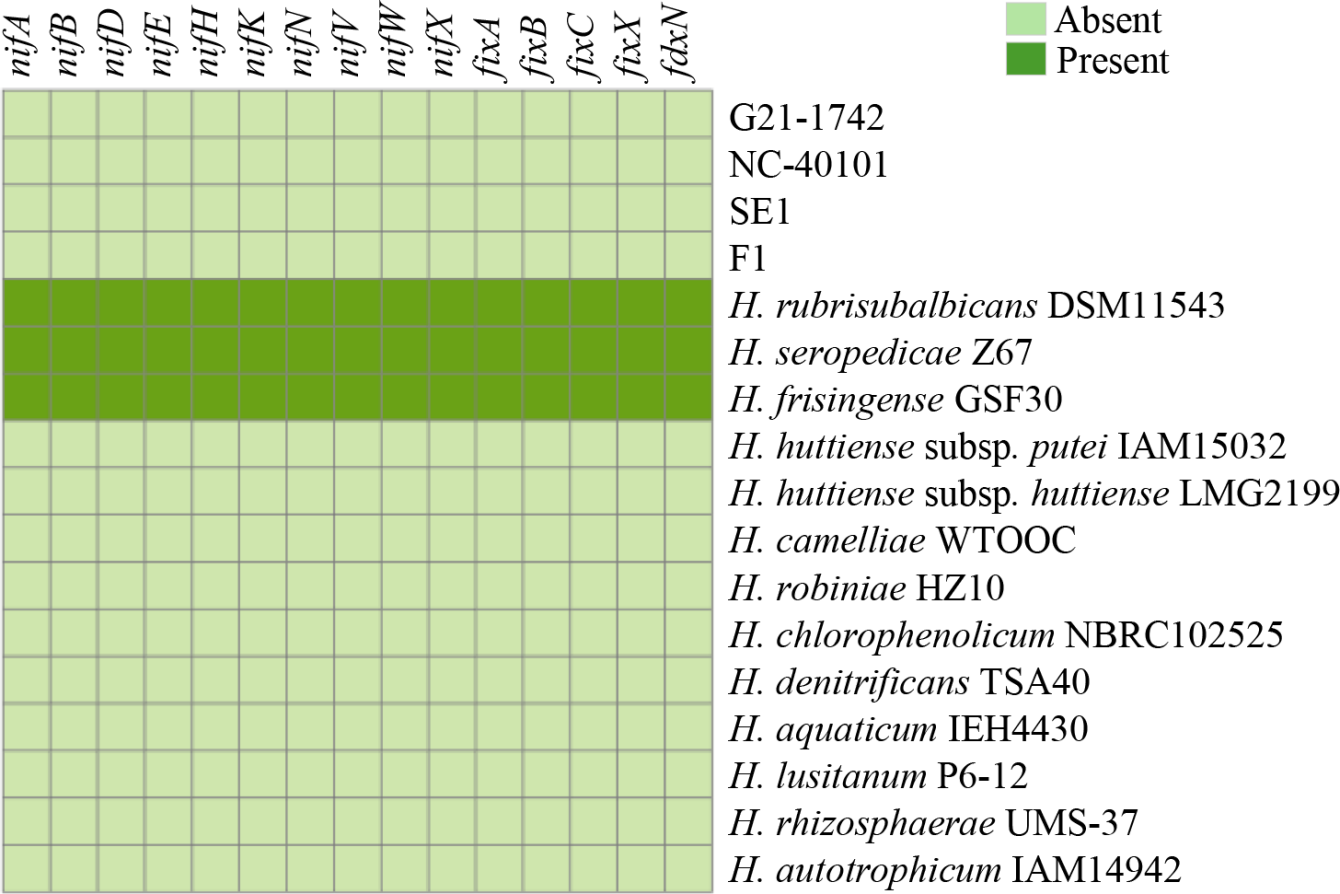
Presence/absence matrix of nitrogen fixation genes of strains sequenced in this study and various genomes of *Herbaspirillum* spp.

The genus *Herbaspirillum* is known for its beneficial association with plants including nitrogen fixation [28]. For this reason, we compared the presence/absence of various nitrogen fixation (*nif*) and nitrogenase reductase (*fix*) genes across different genomes of *Herbaspirillum* spp. For the prediction of nitrogen fixation genes, the *nif* gene sequences of *H. seropedicae* strain SmR1 were obtained [28], including *nifA* (WP_013234836.1), *nifB* (WP_006463072.1), *nifH* (WP_013234820.1), *nifD* (WP_013234819.1), *nifE* (WP_013234817.1), nifN (WP_013234816.1), *nifK* (WP_013234818.1), *nifV* (WP_013234805.1), *nifW* (WP_013234806.1) and *nifX* (WP_006463095.1) as well as other accessory genes such as *fixA* (WP_013234807.1), *fixB* (WP_013234808.1), *fixC* (WP_013234809.1) and *fdxN* (WP_013234804.1). These sequences were used as queries for blastp searches against a database formed by all protein sequences from all genomes of *Herbaspirillum* spp. Only hits with ≥50% coverage and ≥70% identity were considered valid. Among the strains analysed, *H. rubrisubalbicans* DSM 11543, *H. seropedicae* SmR1 and *H. frisingense* GSF30 exhibited the presence of the examined *nif* and *fix* genes, which is consistent with previous findings [28] (Fig. 6). Conversely, the fern (G21-1742, NC 40101) and the tomato (SE1, F1) strains along with the type strains of *H. huttiense* showed the absence of these genes. This absence suggests the lack of nitrogen-fixing capability in these strains.

## Conclusion

In summary, multiphasic analyses, including calculation of ANIb based on whole-genome sequences, *is*DDH values, TYGS results, core genome phylogeny, biochemical profiling with the Biolog GENIII MicroPlate system and analysis with MALDI-TOF MS, showed that strains isolated from Boston fern and tomato represent two novel subspecies in the species *H. huttiense*. For these, we propose the names *H. huttiense* subsp. *nephrolepidis* and *H. huttiense* subsp. *lycopersici*.

## Description of *Herbaspirillum huttiense* subsp. *nephrolepidis*

*Herbaspirillum huttiense* subsp. *nephrolepidis* (*ne.phro.le’pi.dis*. N.L. gen. n. *nephrolepidis*, referring to the host, *Nephrolepis exaltata*, from which the strain was isolated).

Cells are rod-shaped, motile with one to three polar flagella (Fig. 1) at one or both poles, Gram-negative, non-fermenting, strictly aerobic, oxidase-, urease- and catalase-positive. Colonies are round, opaque with smooth margins with a diameter of 2–2.5 mm. Cells are flagellar (1–2 flagella) and motile. The strains show consistent metabolic activity in the presence of A-d-glucose, acetic acid, *α*-keto-glutaric acid, *β*-hydroxy-d, l-butyric acid, citric acid, d-arabitol, d-aspartic acid, d-galacturonic acid, d-gluconic acid, d-malic acid, d-saccharic acid, l-fucose, l-galactonic acid, l-lactic acid, l-malic acid, l-pyroglutamic acid, mucic acid, *N*-acetyl-d-glucosamine, *p*-hydroxy-phenylacetic acid and quinic acid.

G+C content of genomic DNA of the type strain is 61%. Cultures were observed to grow at incubation temperatures of 28 and 37, though no growth was observed at 41. The designated strain G21−1742=LMG 33362=NCPPB 4765 was isolated from fronds of *N. exaltata* in North Carolina, USA.

The GenBank/EMBL/DDBJ accession numbers for the 16S rRNA gene and genome sequences of the strain G21-1742 are JAVLSM000000000 and OR004801.1, respectively. The GenBank/EMBL/DDBJ accession numbers for the 16S rRNA gene and genome sequences of the strain NC 40101 are JAVRAA000000000 and PP848216, respectively.

## Description of *Herbaspirillum huttiense* subsp. *lycopersici*

*Herbaspirillum huttiense* subsp. *lycopersici* (*ly.ko.per.si'kon*. noun, referring to the host, *Solanum lycopersici*, from which the strain was isolated).

Cells are rod-shaped, motile with one to three polar flagella (Fig. 1) at one or both poles, Gram-negative, non-fermenting, strictly aerobic, oxidase-, urease- and catalase-positive. Colonies are round, opaque with smooth margins with a diameter of 2–2.5 mm. Cells are flagellar (1–2 flagella) and motile. The strains show consistent metabolic activity in the presence of A-d-glucose, acetic acid, *α*-keto-glutaric acid, *β*-hydroxy-D, l-butyric acid, citric acid, d-arabitol, d-aspartic acid, d-galacturonic acid, d-gluconic acid, d-malic acid, d-mannitol, d-saccharic acid, glycerol, glycyl-l-proline, l-aspartic acid, l-fucose, l-galactonic acid, l-galactonic acid, l-lactic acid, l-malic acid, l-pyroglutamic acid, mucic acid, *N*-acetyl-d-glucosamine, *p*-hydroxy-phenylacetic acid and quinic acid.

G+C content of genomic DNA of the type strain is 62%. Cultures were observed to grow at incubation temperatures of 28 and 37, though no growth was observed at 41.The designated strain SE1=LMG 3361=NCPPB 4764 was isolated from leaves of *Solanum lycopersici* in Florida, USA.

The GenBank/EMBL/DDBJ accession numbers for the 16S rRNA gene and genome sequences of the strain SE1 are JAVLSJ000000000 and EF216332.1, respectively. The GenBank/EMBL/DDBJ accession numbers for the 16S rRNA gene and genome sequences of the strain F1 are JAVRAB000000000 and EF216331, respectively.

## supplementary material

10.1099/ijsem.0.006597 Uncited Supplementary Material 1.

## Abbreviations

ANI: average nucleotide identity
ANIb: average nucleotide identity based on blast
CAGECAT: Comparative Gene Cluster Analysis Toolbox
DDH: DNA–DNA hybridization
*is*DDH: *in silico* DNA–DNA hybridization
iTOL: Interactive Tree Of Life
NA: nutrient agar
T3E: type III effector
TEM: transmission electron microscopy
T3SS: type III secretion system
TYGS: Type (strain) Genome Server

## References

[1] Baldani JI, Baldani VLD, Seldin L, Dobereiner J (1986). Characterization of *Herbaspirillum seropedicae* gen. nov., sp. nov., a root-associated nitrogen-fixing bacterium. Int J Syst Evol Microbiol.

[2] Li X, Bao X, Qiao G, Wang L, Shi C (2022). First study of bacteremia caused by *Herbaspirillum huttiense* in China: a brief research report and literature review. Front Cell Infect Microbiol.

[3] Monteiro RA, Balsanelli E, Wassem R, Marin AM, Brusamarello-Santos LCC (2012). Herbaspirillum-plant interactions: microscopical, histological and molecular aspects. Plant Soil.

[4] Pimentel JP, Olivares F, Pitard RM, Urquiaga S, Akiba F (1991). Dinitrogen fixation and infection of grass leaves by *Pseudomonas rubrisubalbicans* and *Herbaspirillum seropedicae*. Plant Soil.

[5] Schmidt MA, Balsanelli E, Faoro H, Cruz LM, Wassem R (2012). The type III secretion system is necessary for the development of a pathogenic and endophytic interaction between *Herbaspirillum rubrisubalbicans* and Poaceae. BMC Microbiol.

[6] James EK, Olivares FL, Baldani JI, Dobereiner J (1997). *Herbaspirillum*, an endophytic diazotroph colonizing vascular tissue 3Sorghum bicolor L. Moench. J Exp Bot.

[7] Olivares FL, James EK, Baldani JI, Döbereiner J (1997). Infection of mottled stripe disease‐susceptible and resistant sugar cane varieties by the endophytic diazotroph *Herbaspirilium*. New Phytologist.

[8] Ding L, Yokota A (2004). Proposals of *Curvibacter gracilis* gen. nov., sp. nov. and *Herbaspirillum putei* sp. nov. for bacterial strains isolated from well water and reclassification of [*Pseudomonas*] *huttiensis*, [*Pseudomonas*] *lanceolata*, [*Aquaspirillum*] *delicatum* and [*Aquaspirillum*] *autotrophicum* as *Herbaspirillum huttiense* comb. nov., *Curvibacter lanceolatus* comb. nov., *Curvibacter delicatus* comb. nov. and *Herbaspirillum autotrophicum* comb. nov. Int J Syst Evol Microbiol.

[9] Dobritsa AP, Reddy MCS, Samadpour M (2010). Reclassification of *Herbaspirillum putei* as a later heterotypic synonym of *Herbaspirillum huttiense*, with the description of *H. huttiense* subsp. *huttiense* subsp. nov. and *H. huttiense* subsp. *putei* subsp. nov., comb. nov., and description of *Herbaspirillum aquaticum* sp. nov. Int J Syst Evol Microbiol.

[10] Obradovic A, Jones JB, Minsavage GV, Dickstein ER, Momol TM (2007). A leaf spot and blight of greenhouse tomato seedlings incited by a *Herbaspirillum* sp. Plant Dis.

[11] Benitez BN, Poudel M, Jones JB, Harmon CL (2023). First report of *herbaspirillum* sp. causing leaf spots on boston fern (*nephrolepis exaltata*) in florida. Plant dis.

[12] Meier-Kolthoff JP, Carbasse JS, Peinado-Olarte RL, Göker M (2022). TYGS and LPSN: a database tandem for fast and reliable genome-based classification and nomenclature of prokaryotes. Nucleic Acids Res.

[13] Meier-Kolthoff JP, Göker M (2019). TYGS is an automated high-throughput platform for state-of-the-art genome-based taxonomy. Nat Commun.

[14] Timilsina S, Pereira-Martin JA, Minsavage GV, Iruegas-Bocardo F, Abrahamian P (2019). Multiple recombination events drive the current genetic structure of *Xanthomonas perforans* in Florida. Front Microbiol.

[15] Bankevich A, Nurk S, Antipov D, Gurevich AA, Dvorkin M (2012). SPAdes: a new genome assembly algorithm and its applications to single-cell sequencing. J Comput Biol.

[16] Parks DH, Imelfort M, Skennerton CT, Hugenholtz P, Tyson GW (2015). CheckM: assessing the quality of microbial genomes recovered from isolates, single cells, and metagenomes. Genome Res.

[17] Seemann T (2014). Prokka: rapid prokaryotic genome annotation. Bioinformatics.

[18] Pritchard L, Glover RH, Humphris S, Elphinstone JG, Toth IK (2016). Genomics and taxonomy in diagnostics for food security: soft-rotting enterobacterial plant pathogens. Anal Methods.

[19] Richter M, Rosselló-Móra R (2009). Shifting the genomic gold standard for the *prokaryotic* species definition. Proc Natl Acad Sci U S A.

[20] Page AJ, Cummins CA, Hunt M, Wong VK, Reuter S (2015). Roary: rapid large-scale prokaryote pan genome analysis. Bioinformatics.

[21] Stamatakis A (2014). RAxML version 8: A tool for phylogenetic analysis and post-analysis of large phylogenies. Bioinformatics.

[22] Letunic I, Bork P (2007). Interactive Tree Of Life (iTOL): an online tool for phylogenetic tree display and annotation. Bioinformatics.

[23] van den Belt M, Gilchrist C, Booth TJ, Chooi Y-H, Medema MH (2023). CAGECAT: the CompArative GEne Cluster analysis toolbox for rapid search and visualisation of homologous gene clusters. BMC Bioinformatics.

[24] Doonan J, Denman S, Pachebat JA, McDonald JE (2019). Genomic analysis of bacteria in the Acute Oak Decline pathobiome. Microbial Genomics.

[25] Coll NS, Valls M (2013). Current knowledge on the *Ralstonia solanacearum* type III secretion system. Microb Biotechnol.

[26] Kang Y, Jelenska J, Cecchini NM, Li Y, Lee MW (2014). HopW1 from *Pseudomonas syringae* disrupts the actin cytoskeleton to promote virulence in *Arabidopsis*. PLoS Pathog.

[27] Gochez AM, Shantharaj D, Potnis N, Zhou X, Minsavage GV (2017). Molecular characterization of XopAG effector AvrGf2 from *Xanthomonas fuscans* ssp. *aurantifolii* in grapefruit. Mol Plant Pathol.

[28] Pedrolo AM, Matteoli FP, Soares CRFS, Arisi ACM (2023). Comparative genomics reveal the high conservation and scarce distribution of nitrogen fixation nif genes in the plant-associated genus *Herbaspirillum*. Microb Ecol.

