# Supplementary material for: Genomic insights into two new subspecies of Herbaspirillum huttiense strains isolated from diseased foliage in Florida
Source: Int J Syst Evol Microbiol. 2024 Dec 13;74(12):006597. doi: 10.1099/ijsem.0.006597 (PMC11641419; doi:10.1099/ijsem.0.006597)
Supplement: Uncited Supplementary Material 1. [file ijsem-74-06597-s001.pdf]

Supplementary Table 1: Biolog GENIII biochemical profiling for the fern (G21-1742, NC 40101) and tomato strains (SE1, F1) compared with the type strain *H. huttiense* subsp. *huttiense* LMG 2199^T

Carbon Source Utilization:	G21-1742	NC40101	SE1	F1	*H. huttiense* subsp. *huttiense* LMG 2199^T
Dextrin	-	-	-	-	-
D-Maltose	-	-	-	-	-
D-Trehalose	-	-	-	-	-
D-Cellobiose	-	-	-	-	-
Gentiobiose	-	-	-	-	-
Sucrose	-	-	-	-	-
D-Turanose	-	-	-	-	-
Stachyose	-	-	-	-	-
D-Raffinose	-	-	-	-	-
α-D-Lactose	-	-	-	-	-
D-Melibiose	-	-	-	-	-
β-Methyl-D-Glucoside	-	-	-	-	-
D-Salicin	-	-	-	-	-
N-Acetyl-D-Glucosamine	+	+	+	+	+
N-Acetyl-β-D-Mannosamine	-	-	-	-	-
N-Acetyl-D-Galactosamine	-	-	-	-	-
N-Acetyl Neuraminic Acid	-	-	-	-	-
A-D-Glucose	+	+	+	+	+
D-Mannose	-	-	+/-	+/-	+/-
D-Fructose	-	-	-	-	+/-
D-Galactose	+	-	+	-	+
3-Methyl Glucose	-	-	-	-	-
D-Fructose	+/-	+	+	+	+/-
L-Fucose	+	+	+	+	+

L-Rhamnose	-	-	-	-	-
Inosine	-	-	-	-	-
D-Sorbitol	+/-	+	+	+/-	+
D-Mannitol	+	+/-	+	+	+
D-Arabitol	+	+	+	+	+
myo-Inositol	-	-	-	-	
Glycerol	+	+/-	+	+	+
D-Glucose-6-PO4	-	-	+/-	-	
D-Fructose-6-PO4	-	-	+/-	+/-	-
D-Aspartic Acid	+	+	+	+	+
D-Serine	-	-	-	-	-
Gelatin	-	-	-	-	-
Glycyl-L-Proline	+/-	+	+	+	+
L-Alanine	-	+/-	+/-	+	+/-
L-Arginine	-	-	-	-	-
L-Aspartic Acid	+	+/-	+	+	+
L-Glutamic Acid	+	+/-	+	+	+
L-Histidine	-	-	-	-	-
L-Pyroglutamic Acid	+	+	+	+	+
L-Serine	-	-	+/-	+	+/-
Pectin	-	-	-	-	-
D-Galacturonic Acid	+	+	+	+	+
L-Galactonic Acid	+	+	+	+	+
D-Gluconic Acid	+	+	+	+	+
D-Glucuronic Acid	-	-	-	+	+
Glucuronamide	-	-	+/-	-	+
Mucic Acid	+	+	+	+	+
Quinic Acid	+	+	+	+	+
D-Saccharic Acid	+	+	+	+	+
p-Hydroxy-Phenylacetic Acid	+	+	+	+	+
Methyl Pyruvate	-	+/-	+	-	+

D-Lactic Acid Methyl Ester	-	-	-	-	+/-
L-Lactic Acid	+	+	+	+	+
Citric Acid	+	+	+	+	+
α-Keto-Glutaric Acid	+	+	+	+	+
D-Malic Acid	+	+	+	+	+/-
L-Malic Acid	+	+	+	+	+
Bromo-Succinic Acid	+/-	+/-	+	+	+
Tween 40	-	-	-	-	-
Amino- γ-Butyric Acid	-	+/-	+	+	+
α-Hydroxy-Butyric Acid	-	+	+	+/-	+
β-Hydroxy-D,L-Butyric Acid	+	+	+	+	+
α-Keto-Butyric Acid	+/-	+	+/-	+	+
Acetoacetic Acid	-	-	-	+/-	-
Propionic Acid	+/-	+	+/-	+	+
Acetic Acid	+	+	+	+	+
Formic Acid	+/-	+	+	+	+
**Chemical Sensitivity Assays:**					
pH 6	+	+	+	+	+
pH 5	+	+	+	+	+
1% NaCl	+	+	+	+	+
4% NaCl	-	-	+/-	-	-
8% NaCl	-	-	+/-	-	-
1% Sodium Lactate	+	+	+	+	+
Fusidic Acid	+/-	+	+	+	+/-
D-Serine	-	-	+	+	+/-
Troleandomycin	+/-	+/-	+	+	+/-
Rifamycin SV	+	+	+	+	+
Minocycline	+	-	+	+	+/-
Lincomycin	+	+	+	+	+
Guanadine HCl	+/-	+/-	+	+/-	+/-
Niaproof 4	+/-	-	+	+/-	-

Vancomycin	+	+	+	+	+
Tetrazolium Violet	+	+	+	+	+
Tetrazolium Blue	+	+	+	+	+
Nalidixic Acid	+	+	+	+	+
Lithium Chloride	+/-	+/-	+	+/-	-
Potassium Tellurite	-	+/-	+	+	+/-
Aztreonam	+	+	+	+	+
Sodium Butyrate	-	-	+	+/-	-
Sodium Bromate	-	-	-	-	-

4 Negative reactions are shown as (−), positive reactions are shown as (+), and borderline reactions are shown as (+/-).

Supplementary Table 2: tBLASTn results of the type III secretion system genes of *H. rubrisubalbicans* strain M1 with G21-1742, SE1 & *H. seropedicae* strain SmR1

T3SS (*H. rubrisubalbicans* M1)	G21-1742	SE1	*H. seropedicae* SmR1
hrcC	85.96% identity with 87% query coverage	82.94% identity with 88% query coverage	63.62% identity with 82% query coverage
hrpE	94.06% identity with 100% query coverage	80.84% identity with 93% query coverage	44.15% identity with 91% query coverage
hrpD	90.87% identity with 100% query coverage	80.84% identity with 93% query coverage	37.63% identity with 92% query coverage
hrpL	92.57% identity with 100% query coverage	86.37% identity with 100% query coverage	69.94% identity with 85% query coverage
hrcV	93.82% identity with 100% query coverage	90.14% identity with 100% query coverage	77.87% identity with 100% query coverage
hrpQ	94.46% identity with 99% query coverage	81.56% identity with 99% query coverage	45.56% identity with 100% query coverage
hrcN	94.37% identity with 100% query coverage	89.25%, identity with 98% query coverage	83.45% identity with 96% query coverage
hrcQ	100% identity with 100% query coverage	80.4% identity with 100% query coverage	35.38% identity with 92% query coverage
hrcR	91.32% identity with 100% query coverage	88.03% identity with 100% query coverage	75.45% identity with 100% query coverage
hrcS	85.06% identity with 100% query coverage	93.18% identity with 100% query coverage	75.29% identity with 97% query coverage
hrcT	93.56% identity with 100% query coverage	88.24% identity with 99% query coverage	72.87% identity with 97% query coverage
hrcU	83.51% identity with 100% query coverage	88.03% identity with 100% query coverage	52.85% identity with 99% query coverage
hrpB	87.22% identity with 100% query coverage	85.35% identity with 100% query coverage	47.83% identity with 86% query coverage
hrcJ	86.41% identity with 89% query coverage	85.17% identity with 92% query coverage	71.6% identity with 75% query coverage

Supplementary figures

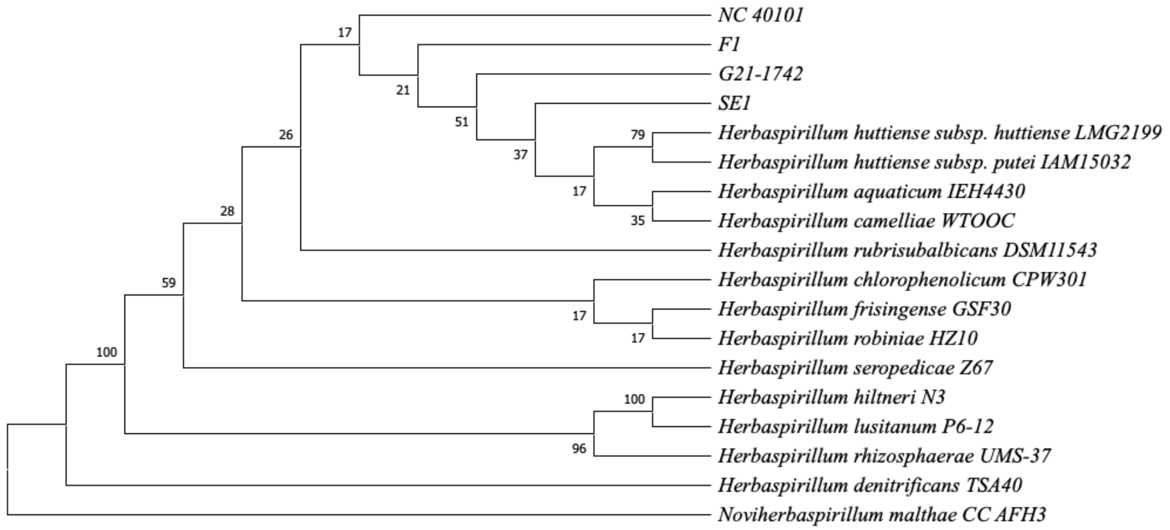

Supplementary. Fig. 1: Maximum likelihood phylogeny of the fern and tomato strains, including type strains of *Herbaspirillum* spp., based on the 16S rRNA region. The tree was rooted at the outgroup.

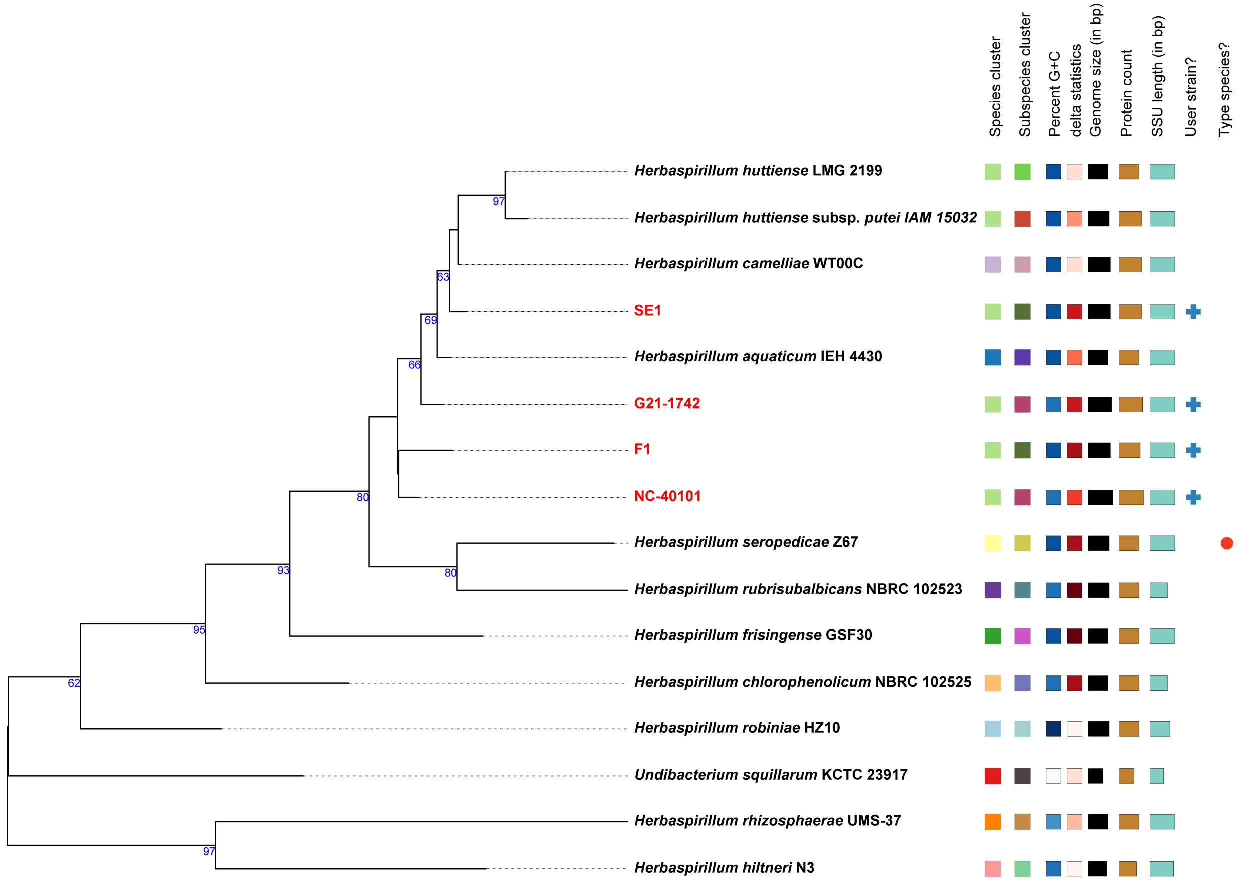

Supplementary Fig. 2: 16S rRNA-based phylogenetic tree highlighting the position of novel fern and tomato strains relative to other *Herbaspirillum* strains. Trees were generated with TYGS server.

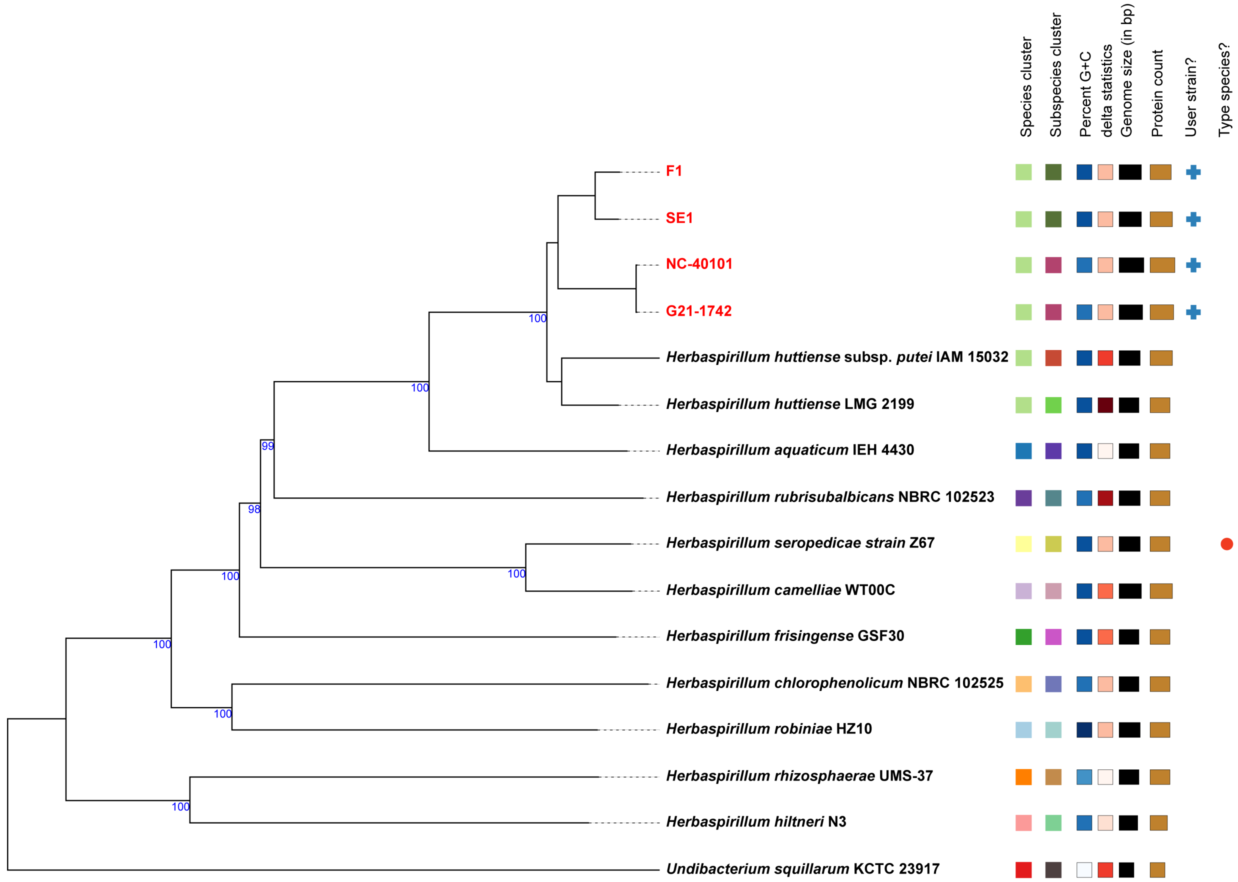

Supplementary Fig. 3: Whole-genome-based phylogenetic tree highlighting the position of novel fern and tomato strains relative to other *Herbaspirillum* strains. Trees were generated with TYGS server.
